# Characterisation of K^+^ Channels in Human Fetoplacental Vascular Smooth Muscle Cells

**DOI:** 10.1371/journal.pone.0057451

**Published:** 2013-02-21

**Authors:** Melissa F. Brereton, Mark Wareing, Rebecca L. Jones, Susan L. Greenwood

**Affiliations:** Maternal and Fetal Health Research Centre, Institute of Human Development, University of Manchester, Manchester Academic Health Sciences Centre (MAHSC), St, Mary's Hospital, Manchester, United Kingdom; Indiana University School of Medicine, United States of America

## Abstract

Adequate blood flow through placental chorionic plate resistance arteries (CPAs) is necessary for oxygen and nutrient transfer to the fetus and a successful pregnancy. In non-placental vascular smooth muscle cells (SMCs), K^+^ channels regulate contraction, vascular tone and blood flow. Previous studies showed that K^+^ channel modulators alter CPA tone, but did not distinguish between effects on K^+^ channels in endothelial cells and SMCs. In this study, we developed a preparation of freshly isolated CPASMCs of normal pregnancy and investigated K^+^ channel expression and function. CPASMCs were isolated from normal human term placentas using enzymatic digestion. Purity and phenotype was confirmed with immunocytochemistry. Whole-cell patch clamp was used to assess K^+^ channel currents, and mRNA and protein expression was determined in intact CPAs and isolated SMCs with RT-PCR and immunostaining. Isolated SMCs expressed α-actin but not CD31, a marker of endothelial cells. CPASMCs and intact CPAs expressed h-caldesmon and non-muscle myosin heavy chain-2; phenotypic markers of contractile and synthetic SMCs respectively. Whole-cell currents were inhibited by 4-AP, TEA, charybdotoxin and iberiotoxin implicating functional K_v_ and BK_Ca_ channels. 1-EBIO enhanced whole cell currents which were abolished by TRAM-34 and reduced by apamin indicating activation of IK_Ca_ and SK_Ca_ respectively. BK_Ca_, IK_Ca_ and SK_Ca_3 mRNA and/or protein were expressed in CPASMCs and intact CPAs. This study provides the first direct evidence for functional K_v_, BK_Ca,_ IK_Ca_ and SK_Ca_ channels in CPASMCs. These cells display a mixed phenotype implicating a dual role for CPASMCs in controlling both fetoplacental vascular resistance and vasculogenesis.

## Introduction

Appropriate control of human placental blood flow is necessary for maximal exchange of oxygen and nutrients to the growing fetus and a successful pregnancy. Placental chorionic plate arteries (CPAs) branch from the umbilical arteries and are likely the primary determinants of fetoplacental vascular resistance as they have similar size characteristics (<500 µm) to systemic resistance vessels [1]. Fetoplacental vascular resistance falls across gestation, indicated clinically by umbilical artery Doppler waveform analysis. Fetoplacental blood vessels lack innervation and respond poorly to potent vasoactive agents of the systemic circulation [1], [2]. The primary mechanism to elicit vasodilation and maintain low vascular resistance throughout gestation is flow-induced nitric oxide (NO) release [3]. A high flow/low resistance circulation is essential to promote sufficient maternal-fetal exchange of oxygen and nutrients. Appropriate regulation of SMC function, and therefore fetoplacental vascular tone and blood flow, is necessary to facilitate maximal exchange of these substances and thereby support fetal growth. However, CPA smooth muscle cell (CPASMC) excitation-contraction coupling is poorly understood and studies are currently hindered by the lack of a suitable single cell model of isolated CPASMCs.

In non-placental vascular SMCs, potassium (K^+^) channels are important in controlling excitation-contraction coupling [4]. K^+^ channels are important determinants of the resting membrane potential in vascular SMCs and are regulated by circulating vasoconstrictors and vasodilators. Membrane depolarisation, resulting from K^+^ channel closure, provides the trigger for opening of voltage-gated Ca^2+^ channels and the subsequent Ca^2+^ influx to promote vasoconstriction [5]. Conversely, K^+^ efflux due to K^+^ channel openings causes membrane hyperpolarisation and therefore vasodilation. Non-placental VSMCs express members from all four K^+^ channel families; K_v_, K_IR_, K_2_P and K_Ca_ [4].

A small number of studies have indirectly assessed the role of K^+^ channels in CPASMC excitation-contraction coupling in normal pregnancy. Using a range of K^+^ channel modulators in the *in vitro* perfused placenta and isolated CPAs, K_v_, K_ATP_, K_2_P and K_Ca_ channels have been implicated in regulating basal and agonist-induced tone [6], [7], [8], [9], [10], [11], [12]. K^+^ channels have also been implicated in the maintenance of the resting membrane potential of CPASMCs around −38 mV and its sensitivity to high external K^+^ [13]. Membrane depolarisation and hyperpolarisation elicited by serotonin (5-HT) and acetylcholine (ACh) respectively, was modulated by charbydotoxin and glibenclamide suggesting the presence of K_Ca_ and K_ATP_ channel conductances in CPASMCs [14]. Previous expression studies demonstrate mRNA and protein for BK_Ca_ and some K_v_ isoforms in whole placental homogenate or intact CPAs [8], [10], [11], [15]. However, functional studies of CPA constriction or relaxation with channel modulators have not distinguished between effects on K^+^ channels expressed in endothelial cells and smooth muscle cells. The ion channel physiology of SMCs from resistance CPAs has yet to be investigated directly.

In this study, we developed a preparation of freshly isolated SMCs from CPAs of normal pregnancy and characterised K^+^ channel currents, mRNA and protein expression using whole-cell electrophysiology, immunocytochemistry and RT-PCR.

## Methods

### Ethical Approval

This work was performed with ethical approval from the North West (Haydock Park) Research Ethics Committee (Ref: 08/H1010/55) and informed written consent obtained for all collected tissue. Term placentas (37–42 weeks gestation; N = 40) were collected within 30 min of delivery (vaginal delivery or elective caesarean section) from women with uncomplicated pregnancies (no evidence of hypertension, FGR or other medical disorders). The investigation conforms to the principles outlined in the Declaration of Helsinki.

### Isolation of chorionic plate arterial smooth muscle cells

Small (150–500 µm internal diameter) CPAs were dissected from placental biopsies using fine dissecting forceps. CPAs were cut into 5 mm lengths and maintained in Ca^2+^ free-dissociation media (DM containing in mM: 120 NaCl, 25 NaHCO_3_, 4.2 KCl, 0.6 KH_2_PO_4_, 1.2 MgCl_2_, 11 Glucose; pH 7.4 for 5 min). CPAs were transferred into a tube containing 1 ml DM containing papain, and DTT (both 1.0 mg/ml) for 20 min at 37°C. The tissue was washed three times in ice-cold Ca^2+^ free-DM and transferred to 0.01 mM Ca^2+^-DM containing; collagenase type 1A and collagenase type F (both 1.0 mg/ml) and incubated at 37°C for 10 min. The vessels were washed three times in ice-cold 0.01 mM Ca^2+^-DM and triturated with a fire-polished glass Pasteur pipette.

### Characterisation of chorionic plate arterial smooth muscle cells

Immunocytochemistry was performed on the cell isolates following methanol fixation to determine; (1) the purity of the isolation technique using SMC markers α-smooth muscle actin (α-SMA; 15 µg/ml; A2547 Sigma-Aldrich), myosin-heavy chain-2 (MHC-2; 1∶250; ab683 Abcam), and the endothelial cell marker CD31 (5 µg/ml; M0823 DAKO), (2) SMC phenotype using the contractile marker h-caldesmon (7.7 µg/ml; C4562 Sigma-Aldrich) and synthetic marker non-muscle myosin heavy chain-B (NMMHC-B; 1∶1000; ab684 Abcam). Endogenous peroxidase activity was quenched with 3% H_2_O_2_ for 10 min. Non-specific binding was prevented by incubation for 30 min with non-immune block (10% goat serum, 2% human serum, and 0.1% Tween 20 in TBS). Primary antibodies were optimised and diluted in non-immune block and incubated overnight at 4°C. Negative controls were performed following substitution of the primary antibody with the corresponding concentration of non-immunised IgG. Antibody binding was detected by application of secondary antibody (biotinylated goat anti-mouse IgG; E0433; DAKO) for 30 min and avidin peroxidase (100 µg/ml in TBS) for a further 30 min. Staining was developed using diaminobenzidine (0.75% solution in TBS) and counterstained with Harris's haematoxylin, dehydrated and then mounted.

SMC marker protein expression was confirmed in intact CPAs. CPA sections were formalin fixed for 24 h, dewaxed, rehydrated, and microwaved for antigen retrieval in 0.01 M sodium citrate (pH 6.0). The subsequent incubation steps were repeated as described above.

### Whole-cell K^+^ current recordings

Spindle-shaped, relaxed SMCs obtained within 4 h of isolation were selected for electrophysiology experiments. Aliquots of the cell suspension (50 µl) were left to settle and attach for 20–30 min before addition of extracellular solution (2 ml). Recordings were made using the whole-cell patch-clamp technique [16]. Haematocrit glass patch pipettes (3–6 MΩ resistance) were pulled using a vertical pipette puller (PC-10, *Narishige*). Voltage protocols were applied using an Axopatch 200B amplifier (Molecular Devices, Sunnyvale, CA, USA) with pCLAMP 10.2 software (Axon Instruments). Cells were voltage clamped at −60 mV and step depolarised from −70 mV to +80 mV for 500 ms in 10 mV increments and repolarised to −40 mV. Membrane capacitance was calculated using manual whole-cell capacitance controls on the Axopatch amplifier. All recordings were performed at room temperature (22–25°C).

Cells were bathed with extracellular solution containing (mM): 140 NaCl, 5 KCl, 1 CaCl_2_, 1 MgCl_2_, 5 HEPES, 10 Mannitol and 5 Glucose (pH 7.3 with NaOH). Patch pipettes were filled with (mM): 120 K-aspartate, 20 KCl, 1 MgCl_2_, 0.5 EGTA, 35 Mannitol and 5 HEPES (pH 7.2 with KOH). K^+^ channel function was assessed by extracellular application of 4-aminopyridine (4-AP; K_v_ inhibitor; 5 mM), tetraethylammonium (TEA; K_Ca_ inhibitor; 5 mM), charybdotoxin (ChTx; BK_Ca_ and IK_Ca_ inhibitor 100 nM), iberiotoxin (IbTx; BK_Ca_ inhibitor; 100 nM), TRAM-34 (IK_Ca_ inhibitor; 10 µM), apamin (SK_Ca_ inhibitor; 100 nM) and 1-EBIO (IK_Ca_ opener; 100 µM). Drug concentrations were chosen according to previous electrophysiology experiments performed in non-placental vascular SMCs [17], [18], [19], [20], [21], [22], [23]. All K^+^ channel modulators were diluted in extracellular solution. Once whole-cell currents had stabilised in control solutions, K^+^ channel modulators were microinjected into the bath solution and currents allowed to approach a new steady state prior to recording. Poor adhesion of the cell isolates to the recording chamber necessitated the use of a static perfusion system and prevented washout of drugs.

### K^+^ channel protein expression in chorionic plate arterial SMCs

Expression of BK_Ca_ (10 µg/ml; APC-107 Alomone Labs) and IK_Ca_ (8 µg/ml; APC-064 Alomone Labs) was assessed in CPASMCs by immunocytochemistry and confirmed in CPA sections.

### K^+^ channel mRNA expression in chorionic plate arteries

Total RNA was extracted from CPAs (approximately 3 cm lengths; diameter <500 µm) using the RNeasy^®^ Fibrous Tissue Mini Kit (QIAGEN^®^, Crawley, UK) according to the manufacturer's instructions, including a DNase incubation step. RNA purity was assessed using spectrophotometric analysis and quantified using a Quant-iT™ Ribogreen^®^ RNA assay kit with reference to rRNA standards (Molecular Probes, Invitrogen). RNA extracted from CPAs isolated from 10 placentas was stored at -80°C. cDNA was generated in duplicate from 25 ng RNA using a Stratagene Affinity Script Multi-temperature cDNA synthesis kit (Agilent, Stockport, UK). Real-time PCR was performed for a housekeeping gene and cDNA pooled if acceptable duplicates using a Stratagene MX3000P system (Agilent) using Brilliant II SYBR^®^ Green Master Mix (Stratagene). Primers used have either been previously been described by our group in chorionic plate arteries; BK_Ca_ (F)5'-AAGCAACGGAATGGAGGCAT-3' (R)5'-CCAGTGAAACATCCCAGTAGAGT-3', or obtained from PrimerBank and optimised for use in this system; IK_Ca_ (F)5'-GCTGCTGCGTCTCTACCTG-3' (R)5'-AAGCGGACTTGATTGAGAGCG-3'; SK_Ca_3 (F)5'-GGCGGATAGCCATGACCTAC-3' (R)5'-CGTGCCGTCCAGAAGAACTT-3'. BLAST searches were performed to ensure primers had no homology with other known gene products. Cycling parameters were: 1 cycle at 95°C for 10 minutes, 40 cycles at 95°C for 30 s (denature), X°C for 1 min (X denotes the annealing temperature specific for each primer set; BK_Ca_; X = 60, IK_Ca_ and SK_Ca_3; X = 61) and 72°C for 1 min (extension). Fluorescence measurements were calculated after each anneal and extension step. A dissociation curve was performed for all PCRs to ensure specificity of the reaction. All PCRs were conducted in duplicate with negative controls on the same plate. cDNA generated from human reference RNA was used as a positive control. PCR products were resolved using 2% agarose gel electrophoresis.

### Chemicals and statistical analysis

General chemicals and pharmacological agents were purchased from Sigma-Aldrich, Poole, Dorset, UK including; collagenase type F, collagenase type 1A, DTT (DL-Dithiothreitol), papain, goat serum, avidin peroxidise, Tween-20, 3,3′-Diaminobenzidine tetrahydrochloride hydrate (DAB), Harris's haematoxylin, 4-AP TEA, 1-EBIO and TRAM-34. Swine serum was obtained from Vector Laboratories Inc., Burlingame, CA, USA. Charybdotoxin, iberiotoxin and apamin were purchased from Alomone Labs, Jerusalem, Israel. Whole-cell currents were normalised to cell capacitance and results represented as mean ± S.E.M (n =  no. cells; N =  no. placentas; statistical evaluation was performed using Wilcoxon-matched pairs signed rank test and Two-way ANOVA with n as the number of cells obtained from a minimum of N = 3 placentas). Current-voltage relationships were constructed from currents measured at 490 ms and significance considered at the P<0.05 level.

## Results

### Characterisation of chorionic plate arterial SMCs

**Figure 1:**
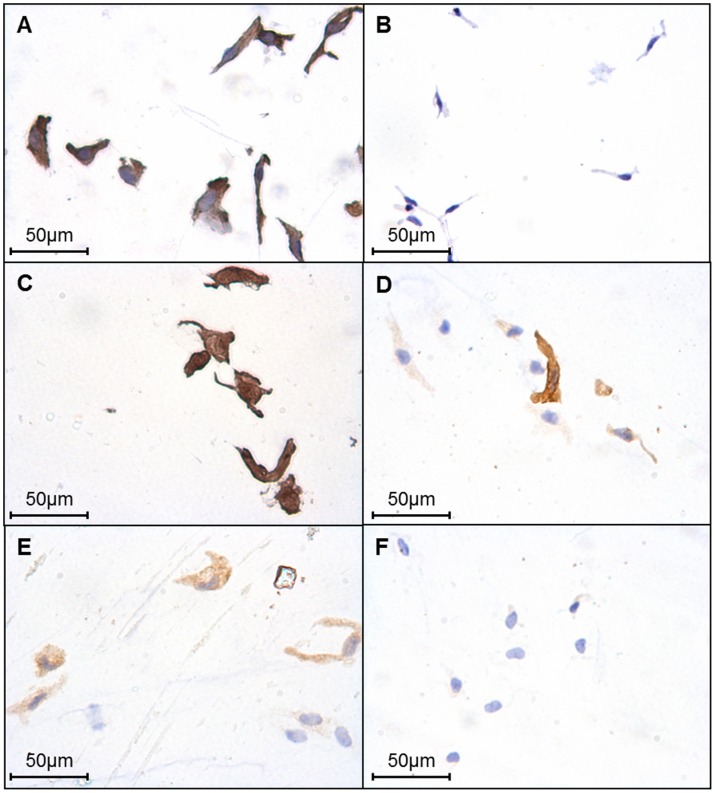
Phenotype characterisation of isolated CPASMCs. Representative examples of immunocytochemistry (**A**) α-smooth muscle actin (α-SMA), (**B**) CD31, (**C**) myosin heavy chain-2 (MHC-2), (**D**) h-caldesmon, (**E**) non-muscle myosin heavy chain-B (NMMHC-B), and (**F**) negative control; non-immune IgG. Positive immunostaining (DAB; brown) and nuclei (haematoxylin; blue).

Cell isolates had a long, oval shaped morphology. They displayed positive immunostaining for the SMC markers α-smooth muscle actin (α-SMA; N = 4; Figure 1A) and myosin heavy chain-2 (MHC-2; N = 3; Figure 1C), and an absence of staining for the endothelial cell marker CD31 (N = 3; Figure 1B), confirming that they were SMCs. Consistent with previous studies in CPAs *in situ* [24], CPASMCs expressed the contractile and synthetic SMC phenotypic markers h-caldesmon (N = 3; Figure 1D) and non-muscle myosin heavy chain-B (NMMHC-B; N = 3; Figure 1E) respectively. Staining for these proteins was variable as cells displayed both positive and negative staining for h-caldesmon and NMMHC-B in the same field of vision (Figure 1 D, E).

**Figure 2:**
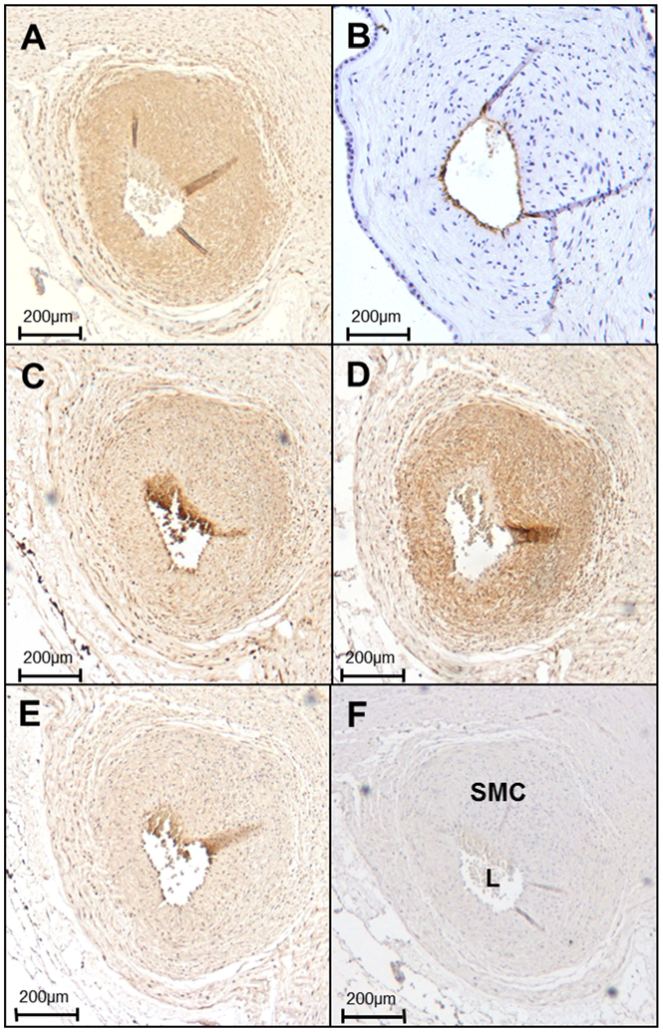
Phenotype characterisation of CPASMCs. Representative examples of immunohistochemistry (**A**) α-smooth muscle actin (α-SMA), (**B**) CD31 (**C**) myosin-heavy chain-2 (MHC-2) (**D**) h-caldesmon, (**E**) non-muscle myosin heavy chain-B (NMMHC-B), and (**F**) negative control; non-immune IgG. Positive immunostaining (DAB; brown) and nuclei (haematoxylin; blue). SMC; smooth muscle cell, L; lumen.

In keeping with isolated SMCs, SMCs of CPA sections expressed proteins that are associated with both contractile, α-SMA (N = 4; Figure 2A), MHC-2 (N = 4; Figure 2C), h-caldesmon (N = 4; Figure 2D) and synthetic NMMHC-B (N = 4; Figure 2E) SMCs. The endothelium was clearly visible with CD31 staining (N-4; Figure 2B). No staining was evident in either CPASMCs (N = 4; Figure 1F) or CPA sections (N = 4; Figure 2F) following substitution of primary antibody with non-immune IgG at an equivalent concentration.

### Characterisation of passive membrane properties and whole-cell current profiles

**Figure 3:**
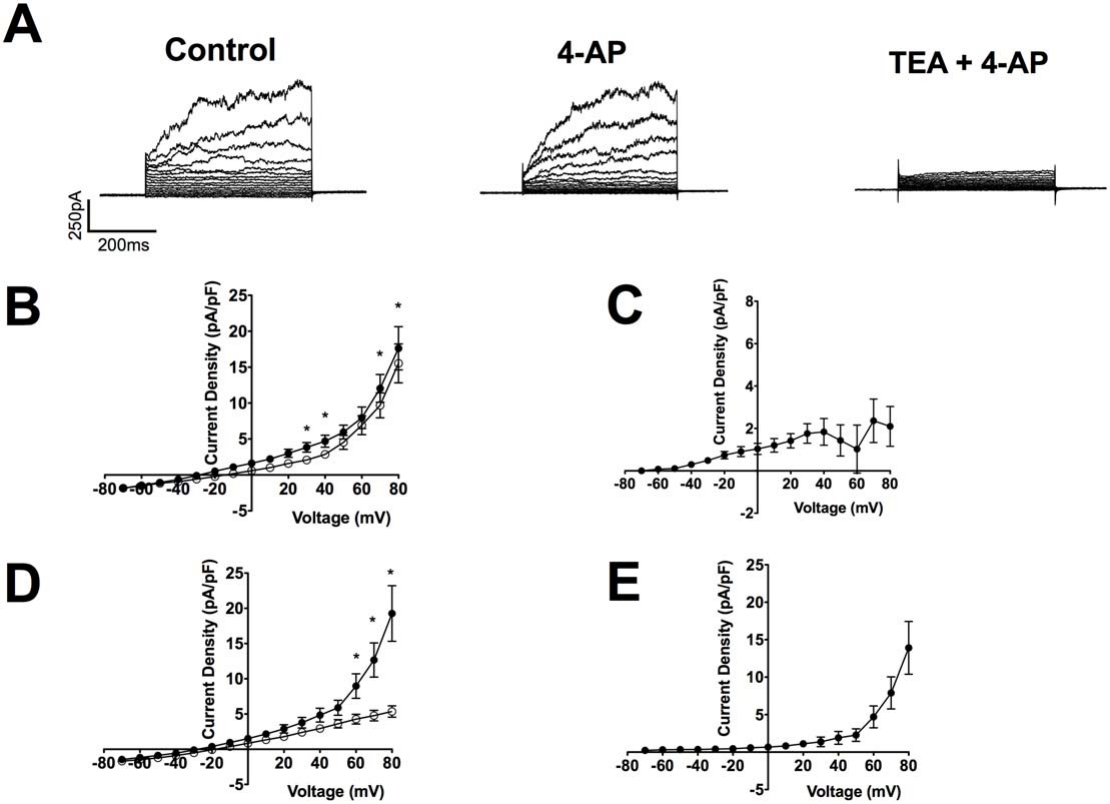
Characterisation of functional voltage-gated (K_v_) and Ca^2+^-activated K^+^ channels (K_Ca_) in CPASMCs. Representative example of the inhibition of outward currents in CPASMCs at negative membrane potentials by the K_v_ blocker 4-aminopyridine (4-AP; 5 mM; **A**) and at depolarised potentials following co-application of the K_Ca_ inhibitor tetraethylammonium (TEA; 5 mM; **A**). Mean current-voltage relationships measured at the end of the 500 ms voltage step ranging from −70 mV to +80 mV were obtained in the absence (•) and presence (○) of 4-AP (**B**; n = 11, N = 8) and TEA alone (**D**; n = 11, N = 6). * indicates currents (mean ± SEM) P<0.05; Two-way ANOVA followed by Bonferroni Post Hoc Test. 4-AP (**C**) and TEA (**E**) sensitive currents were linear and outwardly rectifying respectively.

Membrane capacitance of CPASMCs ranged from 11.0 pF to 41.8 pF (23.2±7.6 pF; mean±SEM; n = 66; N = 24). Step depolarisation from a holding potential of −60 mV to a series of test potentials between −70 mV and +80 mV elicited outward currents with a threshold potential of activation between −40 mV and −10 mV. In the majority of cells (56/66; 85%), currents displayed time-dependent activation, were outwardly rectifying and current magnitude fluctuated at depolarised potentials (e.g. Figure 3A) characteristic of transient openings of BK_Ca_ channels described in SMCs of several vascular beds [17], [18], [19], [20], [21], [25]. These currents were superimposed upon a smaller, time-independent current. In 15% of cells; the smaller current was only recorded under basal conditions. The number of voltage steps displaying fluctuating currents, their magnitude and time course was variable between cells and did not correlate with any apparent visual differences in cell morphology under a light microscope.

### K^+^ channel currents in chorionic plate arterial SMCs

To identify K^+^ channels that may contribute to whole-cell currents in CPASMCs, a pharmacological approach was employed.

The broad-spectrum K_v_ channel blocker 4-AP (5 mM), had a small but significant inhibitory effect on the small time-independent current at potentials between 0 mV and +40 mV (at +40 mV: control 4.7± 0.8 pA/pF; 4-AP 2.9±0.4 pA/pF; P<0.05; Wilcoxon matched-pairs signed rank test; n = 11, N = 8; Figure 3A). Current-voltage relationships measured at the end of the 500 ms voltage step were obtained in the absence (•) and presence (○) of 5 mM 4-AP with significant inhibition of currents evident at +30 mM and +40 mV, and potentials depolarised to +70 mV (P<0.05; Two-way ANOVA followed by Bonferroni Post Hoc Test; Figure 3B). 4-AP sensitive current reversed close to the K^+^ equilibrium potential implicating the presence of K_v_ channel current (Figure 3C). The majority of the outward currents were not affected by 4-AP.

Application of the broad-spectrum K_Ca_ blocker TEA (5 mM) abolished the majority of outward currents in CPASMCs that were insensitive to 4-AP (Figure 3A). TEA preferentially inhibited at potentials positive to +50 mV, with a 66±5% decrease observed at +80 mV (n = 11; N = 6). Current-voltage relationships measured at the end of the 500 ms voltage step were obtained in the absence (•) and presence (○) of TEA alone with significant inhibition of currents evident at potentials depolarised to +60 mV (P<0.05; Two-way-ANOVA followed by Bonferroni Post Hoc Test Figure 3D). TEA-sensitive currents were outwardly rectifying and reversed close to the K^+^ equilibrium potential indicating K_Ca_ channel currents (Figure 3E).

**Figure 4:**
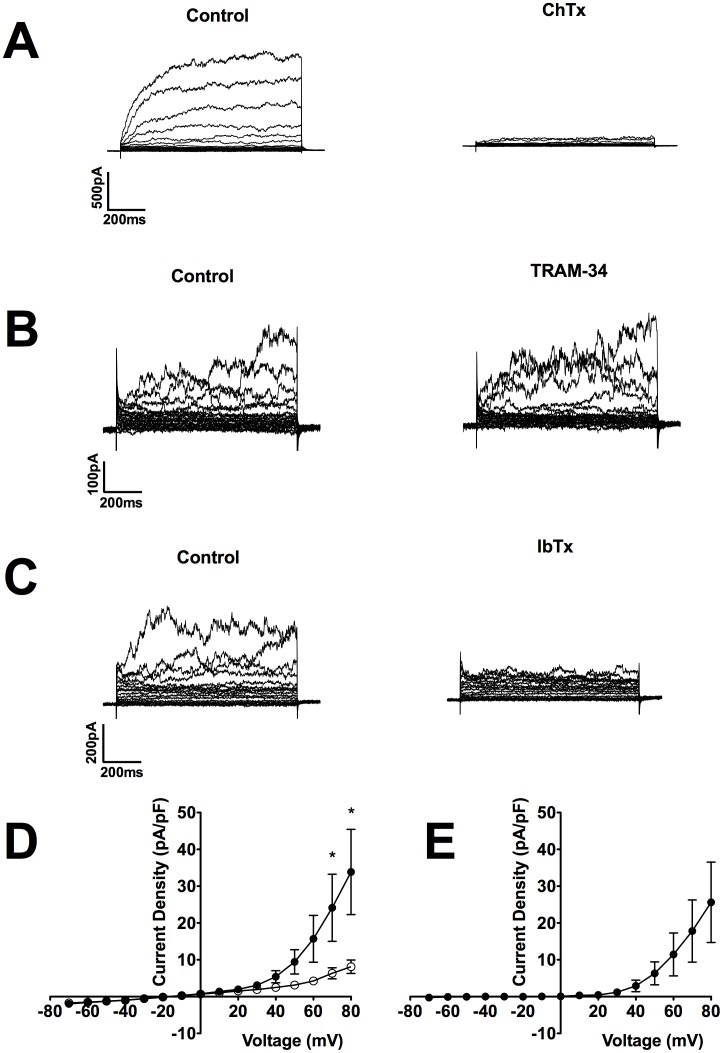
Characterisation of Ca^2+^-activated K^+^ channel isoforms in CPASMCs. Representative example of the inhibition of outward currents by the BK_Ca_ and IK_Ca_ blocker charybdotoxin (ChTx; n = 4; N = 2; 100 nM; **A**), but not the specific IK_Ca_ inhibitor TRAM-34 (n = 3, N = 2; 10µM; **B**). The specific BK_Ca_ blocker iberiotoxin (IbTx; 100 nM; **C**) inhibited outward currents at depolarised potentials. Mean current-voltage relationships measured at the end of the 500 ms voltage step ranging from -70 mV to +80 mV were obtained in the absence (•) and presence (○) of IbTx (**D**; *P<0.05; Two-way ANOVA followed by Bonferroni Post Hoc Test; mean ± SEM; n = 8, N = 4). IbTx-sensitive currents (**E**) were outwardly rectifying.

Identifying the specific K_Ca_ isoform responsible for CPASMC currents utilised application of pharmacological blockers. Inhibition of BK_Ca_ and IK_Ca_ isoforms with ChTx abolished outward currents (n = 4; N = 2; Figure 4A). Selective blockade of IK_Ca_ channels with TRAM-34 was without effect on outward currents (n = 3, N = 2; Figure 4B). Conversely, inhibition of BK_Ca_ channels with IbTx abolished outward currents at +80 mV by 61±12% (n = 10, N = 4; Figure 4C), in common with TEA (Figure 3) and ChTx (Figure 4A). Current-voltage relationships measured at the end of the 500 ms voltage step were obtained in the absence (•) and presence (○) of IbTx with significant inhibition evident at potentials depolarised to +70 mV (P<0.05; Two-way-ANOVA followed by Bonferroni Post Hoc Test; (Figure 4D). IbTx-sensitive currents were outwardly rectifying and reversed close to the K^+^ equilibrium potential indicating that they were mediated by BK_Ca_ channels (Figure 4E).

**Figure 5:**
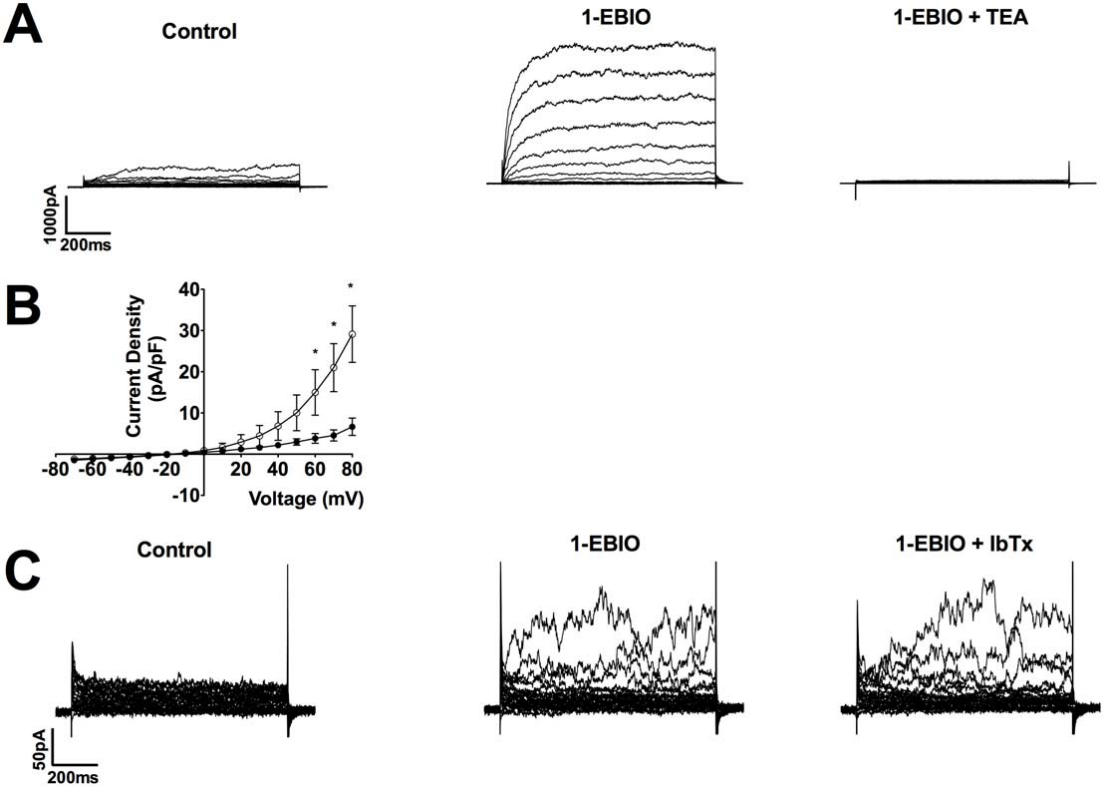
Characterisation of Ca^2+^-activated K^+^ channel openers in CPASMCs. The IK_Ca_ and SK_Ca_ channel activator 1-EBIO (100 µM) increased outwards currents in CPASMCs (**A**). Mean current-voltage relationships obtained at the end of the 500 ms voltage step in the absence (•) and presence (○) of 1-EBIO (**B**; *P<0.05; Two-way ANOVA followed by Bonferroni Post Hoc Test; mean ± SEM; n = 22; N = 10). The 1-EBIO sensitive current was inhibited by the K_Ca_ inhibitor TEA (**A**; 5 mM; n = 2; N = 2), but not the specific BK_Ca_ inhibitor iberiotoxin (IbTx; 100 nM; **C**).

Further analysis of the contribution of K_Ca_ channels to whole-cell currents was performed with extracellular application of 1-EBIO (IK_Ca_ and SK_Ca_ channel activator). 1-EBIO increased whole-cell currents in all cells tested (control: 6.6±2.1 pA/pF; 1-EBIO: 29.1±6.9 pA/pF at +80 mV; P<0.05; n = 22, N = 10; Wilcoxon matched-pairs signed rank test; Figure 5A). Current-voltage relationships measured at the end of the 500 ms voltage step were obtained in the absence (•) and presence (○) of 1-EBIO with significant activation evident at potentials depolarised to +60 mV (P<0.05; Two-way-ANOVA followed by Bonferroni Post Hoc Test; Figure 5B). Tail-currents were observed in 2 out of 22 recordings following 1-EBIO application (see Figure 5A) characteristic of K_v_ channels. However, the low incidence of these currents prevented further analysis. Abolition of the 1-EBIO response with TEA confirmed this increase in whole-cell current was mediated by a K_Ca_ channel (Figure 5A). Experiments to determine the K_Ca_ isoform responsible for 1-EBIO-sensitive current excluded a role for BK_Ca_ as selective blockade of these channels with IbTx in the continued presence of 1-EBIO, had no effect on the magnitude of outward currents (1-EBIO: 15.2±11.2 pA/pF; 1-EBIO + IbTx: 14.2±9 pA/pF at +80 mV; P>0.05; Wilcoxon matched-pairs signed rank test; n = 3, N = 3; Figure 5C).

**Figure 6:**
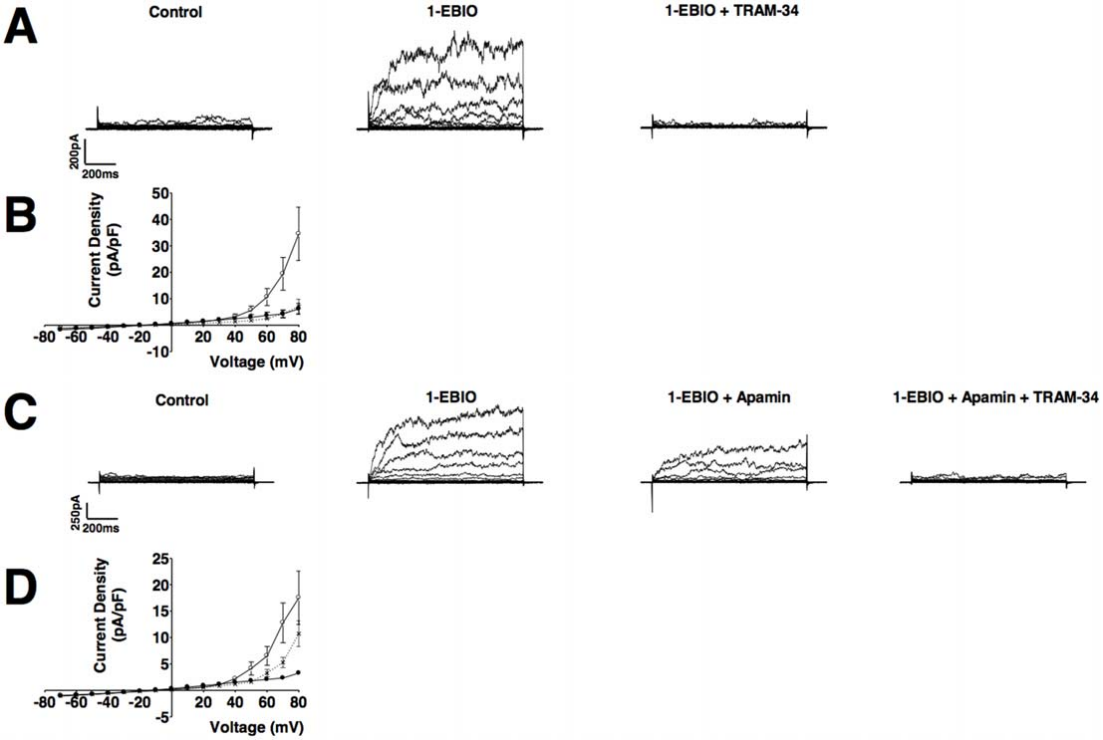
Functional intermediate-conductance (IK_Ca_) and small-conductance (SK_Ca_) Ca^2+^-activated K^+^ channels in CPASMCs. The specific IK_Ca_ inhibitor TRAM-34 (10 µM) abolished 1-EBIO sensitive currents (**A**). Mean current-voltage relationships obtained at the end of the 500 ms voltage step under control conditions (•), and following application of 1-EBIO (○) and TRAM-34 in the continued presence of 1-EBIO (x) (mean±SEM; n = 7; N = 4; **B**). The specific SK_Ca_ inhibitor apamin (100 nM) partially inhibited 1-EBIO sensitive currents and the remaining current was abolished by the subsequent addition of TRAM-34 (**C**). Mean current-voltage relationships obtained at the end of the 500 ms voltage step under control conditions (•), and following application of 1-EBIO (○) and apamin in the continued presence of 1-EBIO (x) (mean±SEM; n = 7; N = 3; **D**).

Selective inhibition of IK_Ca_ isoforms with TRAM-34 (n = 7, N = 4) reduced 1-EBIO sensitive currents (1-EBIO: 34.5±10.0 pA/pF; 1-EBIO + TRAM-34: 7.1±2.7 pA/pF at +80 mV; P<0.05; Wilcoxon matched-pairs signed rank test; n = 7, N = 4; Figure 6A). Current-voltage relationships measured at the end of the 500 ms voltage step were obtained in the absence (•) and presence of 1-EBIO (○) and presence of 1-EBIO with TRAM-34 (x). Addition of TRAM-34 following 1-EBIO application restored the current voltage relationship to the basal level (Figure 6B). In addition, inhibition of SK_Ca_ isoforms with apamin reduced 1-EBIO sensitive currents (+80 mV from 17.5±5.0 pA/pF to 10.7±2.4 pA/pF; P<0.05; Wilcoxon matched-pairs signed rank test; n = 7; N = 3 Figure 6C). The remaining currents were blocked by TRAM-34. Current-voltage relationships measured at the end of the 500 ms voltage step were obtained in the absence (•) and presence of 1-EBIO (○) and presence of 1-EBIO with apamin (x) (Figure 6D).

### K_Ca_ channel expression in chorionic plate arteries

**Figure 7:**
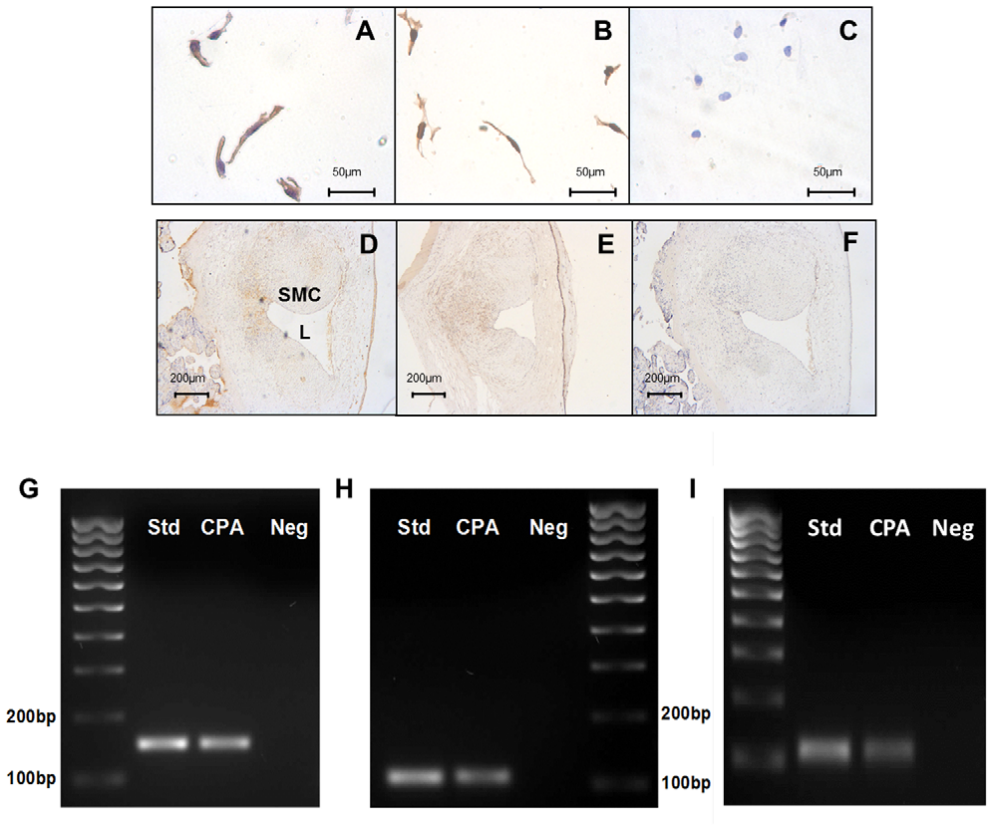
mRNA and protein expression of K_Ca_ channels in CPAs. Isolated CPASMCs expressed BK_Ca_ (**A**) and IK_Ca_ (**B**) protein. BK_Ca_ (**D**) and IK_Ca_ (**E**) protein was localised to the SMCs in intact CPA sections. Negative control (**C**) and (**F**); substitution of primary antibody with non-immune IgG. RT-PCR detection of BK_Ca_ (**G**) IK_Ca_ (**H**) and SK_Ca_3 (**I**) transcripts in human reference cDNA (Std), chorionic plate artery (CPA). Negative (Neg) is omission of cDNA. Amplicon lengths; BK_Ca_ 147, IK_Ca_ 102 and SK_Ca_3 106. SMC; smooth muscle cell, L; lumen.

Protein expression of the pore-forming α-subunits of BK_Ca_ and IK_Ca_ was evident in isolated CPASMCs (N = 3; Figure 7A, B) and intact CPA sections (N = 3; Figure 7D, E). Non-specific staining in either isolated SMCs (N = 4; Figure 7C) or CPA sections (N = 4; Figure 7F) was not observed following substitution of primary antibody with non-immune IgG at an equivalent concentration. mRNA for BK_Ca_ (Figure 7G, 32 ± 1; CT values median ± IQR), IK_Ca_ channels (Figure 7H, 35±2; CT values median ± IQR) and SK_Ca_3 (Figure 7I, 33±2; CT values median ± IQR) was also expressed in intact CPA sections.

## Discussion

Currently, no studies have systematically investigated K^+^ channel activity in SMCs of resistance arteries in the human placenta that regulate fetoplacental blood flow. In this study, a method was developed to isolate SMCs from placental chorionic plate arteries with size characteristics of resistance vessels for whole cell recording to characterise K^+^ currents. We showed that the cells express several K^+^ currents that, on the basis of their pharmacology, could be attributed to K_v_, BK_Ca_, IK_Ca_, and SK_Ca_ channels. The contribution of these channels was supported by their mRNA and/or protein expression in intact arteries and in SMCs after isolation.

The isolation protocol produced a high yield of relaxed CPASMCs, which displayed positive immunostaining for SMC markers and negative staining for an endothelial cell marker. Phenotypic characterisation of CPASMCs confirmed our previous electron microscopy studies of the native artery [24]; isolated CPASMCs and intact CPAs displayed a mixed phenotype, expressing markers of both contractile (h-caldesmon) and synthetic (NMMHC-B) SMC phenotypes. The h-caldesmon and NMMHC-B staining intensity was variable between the isolates suggesting a range of intermediate phenotypes between purely contractile and synthetic. The presence of synthetic SMCs in CPAs, which are important in controlling vasculogenesis, is consistent with the fetal origin of the tissue [26]. A high incidence of synthetic relative to contractile SMCs will impact upon the response of CPAs to modulators of vascular tone and may explain why these vessels are relatively unresponsive to potent vasoactive agents of the systemic circulation [1].

The major K^+^ currents expressed in CPASMCs under basal conditions were investigated. Whole-cell currents comprised a linear, time-independent component and an outwardly rectifying component characterised by the presence of fluctuating currents at depolarised potentials. Current magnitude was variable between cells and was not related to cell size or obvious differences in cell morphology; only relaxed SMCs with a defined membrane were used for electrophysiology experiments. Variability in the current profiles could relate to their phenotype (i.e. synthetic/contractile). However, linking current profiles to SMC phenotype necessitates staining cells that have been recorded which was beyond the scope of this initial investigation to screen K^+^ currents in CPASMCs for the first time.

A pharmacological approach was employed to identify the K^+^ channels responsible for outward currents in CPASMCs. Voltage-gated K^+^ channels (K_v_) were initially targeted given the previous expression and functional data demonstrating a role for these channels in modulating basal and agonist-induced tone in the perfused placenta and isolated CPAs using the K_v_ channel blocker 4-AP [8], [10], [11]. In CPASMCs, 4-AP inhibited currents at relatively negative membrane potentials. This suggests K_v_ channels may be important at physiological membrane potentials and is consistent with 4-AP enhancing basal tone in the intact vessel [8]. The precise K_v_ channel isoforms responsible for 4-AP sensitive currents in CPASMCs remains unknown but previous studies have demonstrated K_v_1.5 [27], K_v_7.4 [28] and K_v_9.3 [29] protein expression in the SMCs of intact CPA sections. Microelectrode impalement studies have demonstrated that the membrane potential of CPASMCs in the intact vessel is approximately −38 mV and is sensitive to high external K^+^ [13]. Membrane depolarisation and hyperpolarisation elicited by serotonin (5-HT) and acetylcholine (ACh) respectively, was modulated by ChTx and glibenclamide suggesting the presence of K_Ca_ and K_ATP_ conductances in CPASMCs [14]. However, it remains to be determined in isolated CPASMCs, whether K_v_ channels *per se* and specifically which isoforms contribute to maintaining the resting membrane potential and are therefore directly responsible for 4-AP induced alterations in basal tone.

Ca^2+^-activated K^+^ channels were next targeted given previous studies demonstrating a role for these channels in modulating fetoplacental vascular tone [8], [10], [11]. The marked inhibition by TEA (K_Ca_ inhibitor), charybdotoxin (BK_Ca_ and IK_Ca_ inhibitor), iberiotoxin (BK_Ca_ inhibitor) but not TRAM-34 (IK_Ca_ inhibitor) suggests that BK_Ca_ channels are responsible for the majority of outward currents in CPASMCs under the recording condition used in the present study. Expression of BK_Ca_ mRNA and protein was confirmed in CPAs and protein localised to the SMCs. In common with non-placental vascular SMCs [17], [18], [19], [20], [21], the large single-channel conductance of BK_Ca_ likely accounts for the characteristic noise and fluctuating currents observed at depolarised potentials in CPASMCs. BK_Ca_ activity in the present study likely reflects membrane depolarisation induced Ca^2+^ entry given the low intracellular Ca^2+^ recording concentrations employed (0.5 mM [EGTA]_i_; predicted [Ca^2+^]_i_ ∼10 nM). Compared to pulmonary resistance arterial SMCs which similarly carry deoxygenated blood but express primarily K_v_ channels, the predominance of K_Ca_ over K_v_ channels in CPASMCs is interesting. Extrapolation of this *in vitro* finding to the physiological situation in the intact vessel or whole placenta where there is a complex ionic environment should be made with caution. However, together with previous observations demonstrating functional BK_Ca_ channels in the whole-perfused placenta, intact artery and large diameter CPASMCs using iberiotoxin and charybdotoxin [8], [10], [11], this study implicates a role for BK_Ca_ channels in CPASMC excitation-contraction coupling.

Further analysis of the role of K_Ca_ channels in CPASMCs demonstrated IK_Ca_ currents. Application of the SK_Ca_ and IK_Ca_ activator 1-EB1O significantly increased outward currents. 1-EBIO sensitive currents were reduced by TEA (K_Ca_ inhibitor), TRAM-34 (IK_Ca_ inhibitor), but unaffected by the BK_Ca_ inhibitor iberiotoxin indicating functional IK_Ca_ channels. In non-placental blood vessels, immunohistochemical and electrophysiological studies do not implicate IK_Ca_ channel expression or function in SMCs, rather, they are localised to the endothelium [23], [30], [31], [32], [33]. Electrophysiology experiments in SMCs isolated from the rat hepatic artery [32] and mouse portal vein [23] failed to observe an effect of 1-EBIO on whole-cell K^+^ currents. This is in marked contrast to CPASMCs where 1-EBIO consistently increased currents at +80 mV by over 400%. The effect of 1-EBIO is strongly Ca^2+^ dependent [34] and stimulation of IK_Ca_ usually requires high intracellular Ca^2+^ concentrations or addition of the Ca^2+^ ionophore ionomycin. Why 1-EBIO is causing significant current activation in CPASMCs given the low recording intracellular Ca^2+^ concentration in this study is unclear. Similarly, the apparent time-dependent activation of the 1-EBIO-sensitive current is inconsistent with the voltage-independent nature of IK_Ca_ channels. However, 1-EBIO-sensitive currents will be modulated by other underlying ion conductances native to these cells. In addition, kinetic analyses were not performed to quantify differences in current profiles following 1-EBIO application given the inherent variability in outward currents recorded. The data demonstrating abolition of 1-EBIO-activated currents with TRAM-34, together with those demonstrating IK_Ca_ mRNA and protein expression, provides strong evidence for the presence of IK_Ca_ channels in CPAs and their localisation to SMCs.

1-EBIO can also activate SK_Ca_ channels. It was therefore investigated whether the SK_Ca_ inhibitor apamin had any effect on 1-EBIO sensitive currents. Application of apamin at a concentration that inhibits both SK_Ca_ isoforms [35] reduced 1-EBIO sensitive currents but to a lesser extent than TRAM-34. In common with IK_Ca_ channels, SK_Ca_ are thought to predominantly localise to the endothelium where they participate in the EDHF response [30], [36]. Therefore, functional SK_Ca_ channels in CPASMCs were unexpected and may be mediated by the SK_Ca_3 isoform given mRNA expression for the pore forming α-subunit in the intact artery. Protein expression of SK_Ca_3 remains to be determined in the intact CPA and CPASMCs. The functional significance of SK_Ca_3 channels in CPASMCs is not known but they may be important in dampening excitation and promoting vasodilation following a rise in intracellular Ca^2+^ in common with BK_Ca_ channels.

It is evident that CPASMCs are a heterogeneous population of cells; they express diverse K^+^ channel currents with varying magnitude and activation profiles, and display a mixed phenotype with both contractile and synthetic characteristics. In other vascular SMCs, a close relationship exists between phenotype and K^+^ channel expression [37], [38], [39]. SMCs with a contractile phenotype predominantly express BK_Ca_ and K_v_ channels which are important in mediating excitation-contraction coupling [18], [22], [25], [40], [41], [42], Conversely, synthetic SMC express IK_Ca_ channels that control proliferation and migration of SMCs during vasculogenesis by promoting Ca^2+^ entry and activation of Ca^2+^-dependent growth factors [38], [43], [44], [45]. The mixed phenotype of CPASMCs with both contractile and synthetic characteristics may account for the functional expression of BK_Ca_, K_v_ and IK_Ca_ channels. BK_Ca_ and K_v_ channel activity in CPASMCs are important in controlling excitation-contraction coupling under basal and agonist-stimulated conditions as pharmacological modulation of these channels alters CPA tone in the intact vessel and perfused placenta [8], [10], [11]. The physiological significance of IK_Ca_ channels in CPASMCs is unexplored. Similarly, whether the existence of this channel in CPASMCs is linked to the synthetic phenotype remains to be confirmed. In addition to a putative role in regulating fetoplacental vascular tone, IK_Ca_ channels may play an important role in controlling CPASMC proliferation and placental vasculogenesis throughout gestation. Further studies are required to directly correlate whole-cell K^+^ currents and the contribution from specific channels with the expression of SMC phenotypic markers.

In conclusion, this study provides the first direct evidence for K_v_, BK_Ca_ IK_Ca_ and SK_Ca_ channel currents in CPASMCs. These cells display a mixed phenotype implicating a dual role for CPASMCs in controlling both fetoplacental vascular resistance and vasculogenesis throughout pregnancy.
